# Generative AI-Driven Discovery of Next-Generation Electrolytes for Alkali Metal Batteries

**DOI:** 10.1021/acs.jcim.6c00135

**Published:** 2026-03-13

**Authors:** Rafiuzzaman Pritom, Md Mahbubul Islam

**Affiliations:** † Department of Mechanical Engineering, 2954 Wayne State University, Detroit, Michigan 48202, United States

## Abstract

Recent advances in artificial intelligence (AI) are revolutionizing materials science by unlocking unprecedented capabilities in designing novel compounds and accurately predicting their properties. Among these, graph-based machine learning (ML) algorithms have garnered significant attention for their ability to capture complex atomic interactions and use them as effective descriptors. In this study, we integrated state-of-the-art generative AI (Gen AI) and ML techniques with quantum mechanical calculations to discover novel next-generation electrolytes for alkali metal batteries. We developed a Generative Adversarial Network (GAN) framework incorporating a graph-based generator and discriminator models to generate novel electrolyte candidates. The GAN model was trained on a subset of approximately 1 million molecules from the GDB-11 database, which enabled the generation of 30,000 unique and chemically valid molecules. Concurrently, a Message Passing Neural Network (MPNN) model was trained for property prediction by utilizing the QM9 dataset. Using the trained MPNN model, we predicted the properties of the newly generated molecules and screened the candidates based on the criteria of negative standard enthalpy of formation and a wide HOMO-LUMO gap. First-principles density functional theory (DFT) calculations were conducted for additional screening and to evaluate key thermodynamic and electrochemical properties, including standard enthalpy of formation, oxidation potential, and reduction potentials. Finally, a set of 26 promising candidates was acquired with outstanding electrochemical characteristics. Our findings demonstrate the potential of AI-driven approaches to discover high-performance, stable, and efficient electrolytes as promising alternatives to conventional organic electrolytes for next-generation energy storage systems.

## Graphical abstract

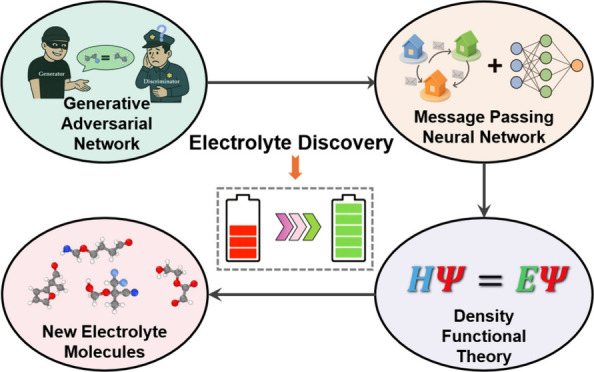

## 1 Introduction

The development of advanced battery technologies highly depends on the discovery of efficient, safe, and chemically stable electrolyte materials. However, the design space for electrolyte molecules is very extensive and multidimensional, which involves trade-offs between properties such as electrochemical stability, ionic conductivity, viscosity, dielectric constant, and compatibility with electrode materials.^1^ Traditional experimental and computational approaches, while foundational, are often too slow and resource-intensive to keep pace with the urgent demand for next-generation energy storage solutions.^2,3^ In this context, machine learning (ML) has emerged as a powerful tool to accelerate electrolyte discovery by learning complex structure–property relationships from existing data and enabling high-throughput screening of candidate molecules.^4–6^ The integration of ML into electrolyte research offers a scalable, cost-effective alternative to conventional trial-and-error methods that creates substantial opportunities for researchers to navigate chemical space more efficiently and identify promising candidates.

Nowadays, ML has garnered substantial attention in material research due to its prediction capability of various properties, including physical, chemical, and electrochemical properties of molecules relevant to materials science. ML models rely on data-driven learning to estimate a vast number of key atomic and molecular properties such as solubility, ionic conductivity, redox potential, thermodynamic stability, viscosity, HOMO–LUMO gap, electrochemical window, and other electronic and thermodynamic properties across diverse molecular systems. For example, Tayyebi et al.^7^ used Random Forest (RF) and multiple Linear Regression (LR) models to predict the aqueous solubility of various components using cheminformatics methods and molecular descriptors and fingerprints as the chemical representation methods. In one study, a deep neural network (DNN), trained on 406 unique and chemically diverse ionic liquids (IL), was used on the experimentally measured and published data to construct rapid and accurate predictions of the conductivity of ILs.^8^ In another study, a multi-component framework, consisting of ML and density functional theory (DFT) calculations, was developed and applied to predict the redox potential, HOMO and LUMO energies of various organic molecules.^9^ Three different ML models—artificial neural networks (ANN), gradient-boosting regression (GBR), and kernel ridge regression (KRR)—were used, and KRR exhibited the highest accuracy in terms of redox potential prediction capability. A data-driven machine learning approach was employed by Liang and Zhang^10^ to predict the thermodynamic phase stability of lead-free halide double perovskites, where they utilized a dataset of 469 A_2_B'BX_6_ compounds with DFT-calculated convex hull energy values and 24 elemental features derived from the periodic table. To accelerate the design of deep eutectic solvents (DESs), various ML models, support Vector Machine (SVR), feed forward neural network (FFNN), and categorical boosting (CatBoost), were developed using COSMO-RS-derived σ-profiles to accurately predict viscosities across temperatures and molar ratios, based on one of the largest existing datasets covering over 670 DESs.^11^ A set of polyacrylic aromatic hydrocarbon molecules was analyzed using DFT calculations to investigate structural factors influencing HOMO–LUMO gaps and an ML model was developed that accurately predicts these gaps with an average absolute error of just 0.19 eV compared to DFT calculations.^12^ Manna et al.^13^ proposed a combined supervised and unsupervised ML framework to efficiently identify solvent electrolytes with optimal electrochemical windows (ECW), and their approach achieved higher accuracy than DFT and reduced the solvent search space into a small number of clusters to accelerate experimental screening for metal-ion battery applications.

Traditional ML techniques in molecular science are often contingent on predefined descriptors based on known molecular properties. Recently, structural descriptors, derived directly from molecular topology, have gained popularity for their ability to provide enriched chemical insights. Previous studies have already shown that graph-based deep learning models can outperform classical ML models.^14^ This shift has brought graph neural network (GNN) to the forefront, as they excel at learning from molecular structures represented as graphs.^15^ As a result, GNNs enable more accurate and versatile predictions of molecular properties across a wide range of research areas. A thorough investigation by John et al.^16^ demonstrated that the message-passing neural network (MPNN), a message-passing-based GNN model, can accurately predict the electronic properties of large organic photovoltaic molecules without relying on optimized 3D geometries, thereby enabling more practical high-throughput screening. Graph convolutional neural network (GCNN) has been reported to offer a robust approach to predict material properties, extract design insights, and approximate DFT results for a wide variety of inorganic crystal structures and compositions.^17^ To improve molecular property prediction and facilitate new material discovery, Chen et al.^18^ introduced another GNN framework that combines graph embeddings, descriptor vectors, and theoretical models and can acquire higher prediction accuracy on public datasets and successfully identify promising drug candidates. An equivariant GNN model was developed to accelerate ab initio molecular dynamics (AIMD) simulations for two-dimensional materials with diverse atomic connectivities, using only time-evolved atomic coordinates for training.^19^ This model accurately predicts key properties—such as potential energy, kinetic energy, entropy, and interatomic force variations—for systems like g-CN, WTe_2_, and their heterostructures, which creates an alternative solution to large-scale AIMD studies by reducing computational cost.

While traditional ML and graph-based models focus on predicting properties of known molecules, generative artificial intelligence (Gen AI) has introduced a paradigm shift by enabling the generation of entirely new molecular structures.^20^ The ability to generate novel molecular structures has revolutionized chemical research, paving the way for the exploration of previously inaccessible regions of chemical space. Recently, significant attention has been directed toward the application of advanced Gen AI techniques such as variational autoencoders (VAEs)^21^ and generative adversarial networks (GANs)^22^ in diverse fields including drug design, materials discovery, catalyst development, battery electrolyte optimization, and so on. For instance, a GAN model has been implemented for the efficient generation of new hypothetical inorganic materials, where ICSD database was used to train the model, and samples of two million unique molecules were obtained.^23^ The application of GAN in drug discovery was highlighted in a study where a GAN-based framework for the *de novo* design of cannabinoid receptor ligands using molecular fingerprints was developed for the target-specific compound generation.^24^ A VAE-based generative model augmented with a binding energy predictor was implemented to design novel catalysts for the Suzuki cross-coupling reaction.^25^ This study achieved an accuracy (2.42 kcal/mol MAE) and generated 84% valid, novel candidates using latent space optimization. Another study presented an iterative approach that combines DFT calculations with GAN to discover high-performance Rh–Ru alloy catalysts for ammonia synthesis, where DFT-derived reaction energies are used to calculate turnover frequencies (TOFs), train a conditional GAN, and iteratively generate novel alloy surfaces with significantly enhanced catalytic activity.^26^ Makoś et al.^27^ proposed a GAN that was developed to generate transition state guess geometries based on the cartesian coordinates of reactants and products for a range of reaction types, including hydrogen migration, isomerization, and transition metal-catalyzed processes. With improved accuracy and a transferable framework, their model was highly efficient in terms of the prediction of transition states in complex chemical reactions. All of these studies highlight the potential of Gen AI as a powerful tool for accelerating state-of-the-art discovery in material space that can uncover innovative solutions with tailored properties across diverse domains.

This influence of ML and Gen AI has also extended into the field of battery electrolytes, which has opened new opportunities for the discovery of advanced materials with optimized properties. The improvement of the prediction of key physicochemical properties for lithium-ion battery electrolytes, active learning integrated with GNNs was implemented using the LIBE dataset^28^ for training and MPcules^29^ for validation.^30^ By employing uncertainty-based sampling, the model achieved higher predictive accuracy with fewer training samples, improving performance across properties like electronic energy, free energy, and vibrational frequencies. A ML force field framework, named BAMBOO, constructed with a graph equivariant transformer (GET) architecture, was able to perform accurate molecular dynamics (MD) simulations of liquid electrolytes for lithium-ion batteries.^31^ It incorporated ensemble knowledge distillation to enhance stability and a physics-based density alignment method to match experimental properties and achieved high accuracy in the prediction of density, viscosity, and ionic conductivity across diverse solvent and salt compositions. Xie et al.^32^ combined bond-valence methods with GNNs to screen lithium-based solid-state electrolytes and identified a pool of promising candidates possessing high ionic conductivity, while also focusing on key structural features through ablation studies. As ML applications to electrolyte design are still in their early stages, many important areas remain unexplored. In particular, the discovery of entirely new electrolyte organic solvent molecules, those not yet synthesized but with the potential to revolutionize battery technology, continues to be a significant challenge in this field. This motivates us to investigate new strategies for discovering novel electrolyte molecules with promising characteristics that can drive the next generation of battery technologies.

Here, we present a systematic approach to accelerate the discovery of next-generation electrolytes for alkali metal batteries by integrating advanced ML and Gen AI technology with quantum mechanical calculations. We developed a GAN model incorporating GNN-based generator and discriminator models to generate novel electrolyte candidates. Our GAN was trained on one million molecules subset obtained from the GDB-11 database^33^, enabling the generation of 30,000 unique and chemically valid molecules. Additionally, we trained an MPNN-based property prediction model using the QM9 dataset^34,35^ , which contains ∼133,000 molecules with key quantum mechanical properties, including G4MP2 standard enthalpy of formation, GW HOMO energy, and GW LUMO energy, and predicted the properties of the newly generated molecules. Subsequently, we downsized the set of candidates based on the criteria of negative standard enthalpy of formation and a wide HOMO-LUMO gap. The selection process is further refined through computing their HOMO, LUMO, oxidative potential, reduction potential, and standard enthalpy of formation by using first-principles DFT calculations, and finally obtained 26 promising candidates. Overall, this study provides us with the scope for the identification of suitable electrolyte materials possessing tremendous potential for the alkali metal batteries.

## 2 Methodology

This work is organized around three major investigations: (1) generating novel electrolyte molecules using an unsupervised GAN model, (2) predicting the key electrochemical properties of these generated molecules using a supervised MPNN model, and (3) validating and characterizing the most promising candidates through DFT calculations. Figure 1 depicts the detailed workflow of this study.

**Figure 1:**
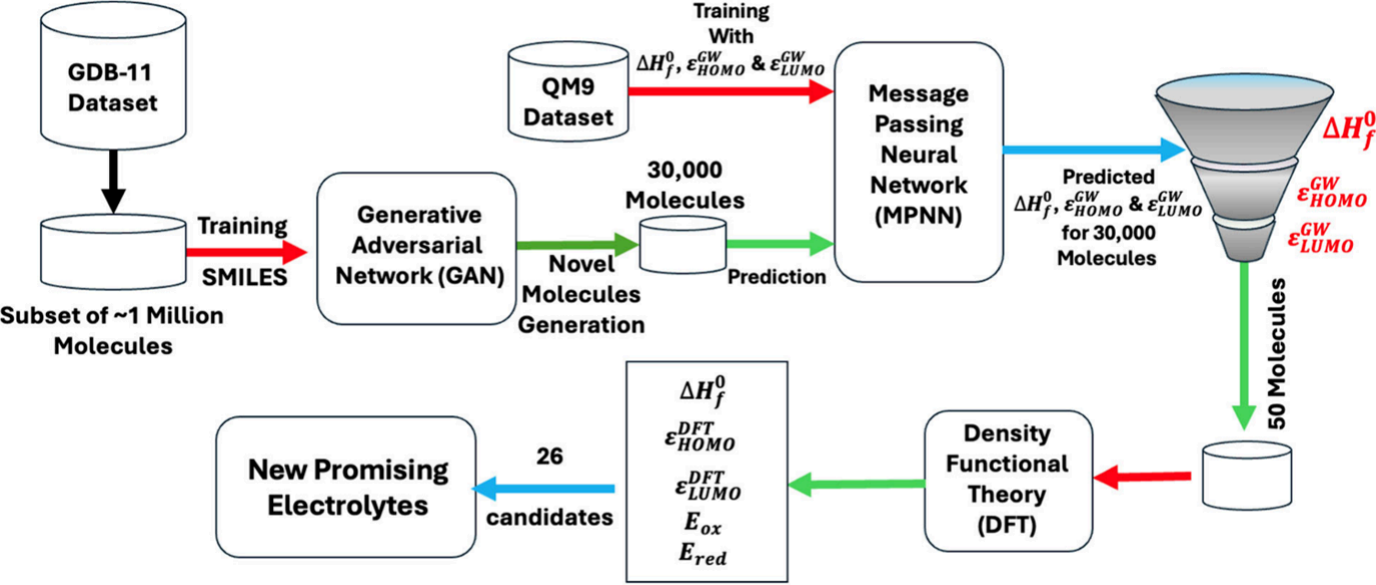
Schematic diagram of the complete workflow in search of new electrolyte materials.

### 2.1 Datasets and Data Pre-Processing

Two distinct datasets were utilized for training the GAN and MPNN models in this study. For the GAN, a subset having one million molecules was selected from the GDB-11 dataset^33^, a systematically generated database comprising stable small organic molecules containing up to 11 heavy atoms, specifically carbon, nitrogen, oxygen, and fluorine. The chosen subset was carefully curated to include a diverse range of molecular structures, covering molecules with between 1 and 11 heavy atoms to ensure both chemical variety and stability. This diversity was essential for facilitating the GAN to learn a broad distribution of molecular patterns and generate novel molecules with structurally valid and chemically meaningful features. For the training of the unsupervised GAN model, only the SMILES^36^ representations of the molecules were considered as input. On the other hand, the QM9 dataset,^34,35^ containing over 133,000 molecules, was selected for training the MPNN model. Similar to the GAN setup, only the SMILES representations of the molecules were used as input. Several electrochemical properties were chosen as target outputs, including the standard enthalpy of formation, HOMO energy, and LUMO energy. The standard enthalpy of formation data was sourced from the work of Narayanan et al.^34^, where they applied the high-accuracy G4MP2 method. The HOMO and LUMO energy values were obtained from the study conducted by Fediai et al.^35^, which provided high-quality quantum mechanical property data for molecules in the QM9 database.

SMILES is a linear text format that does not inherently capture the full topological and chemical information of molecular structures. It is very crucial for ML models to obtain detailed structural information in order to establish correlations between molecular structure and properties. To address this, each SMILES string in the datasets needs to be presented into descriptors that can elucidate the structural characteristics of the molecule. The most efficient way to do this is to convert the molecule into a molecular graph, where atoms are treated as nodes and chemical bonds as edges. The SMILES to graph conversion was performed using the RDKit cheminformatics library. In this process, each atom was assigned a feature vector encoding properties such as atomic number, number of valence electrons, number of bonded hydrogen atoms, and hybridization type (sp, sp^2^, sp^3^). Similarly, each bond was described by features such as bond type (single, double, triple, or aromatic), and bond conjugation. These node and edge features were subsequently organized into tensor formats to serve as inputs for GAN and MPNN. The molecular connectivity within the atoms was interpreted into an adjacency matrix and pair indices in GAN and MPNN, respectively. The adjacency matrix encoded the information regarding the presence and type of bonds between atoms, while the pair indices defined atom-to-atom connections for message passing operation within the GNN framework.

### 2.2 GAN Framework

GAN is a class of generative models that learn to create new data samples resembling a given data distribution through a two-player adversarial game. A schematic representation of a basic GAN framework, illustrating its major components, is shown in Figure 2a. A GAN consists of two neural networks: a generator (G), which maps random noise vectors *z* ∼ *p_z_* (*z*) into synthetic samples, and a discriminator (D), which attempts to distinguish between real samples *x* ∼ *p_data_* (*x*) and generated samples *G*(*z*). The generator tries to produce samples that are indistinguishable from real data, while the discriminator tries to correctly classify the sample as real or fake data. The standard GAN training objective is formulated as a minimax optimization problem shown in eq 1.

1 $$\begin{array}{l} min\\ G \end{array}\begin{array}{l} max\\ D \end{array}E_{x∼p_{data}\left(x\right)}\left[\log\left(D\left(x\right)\right)\right]+E_{z∼p_{z}\left(z\right)}\left[\log\left(1-D\left(G\left(z\right)\right)\right)\right]$$

**Figure 2:**
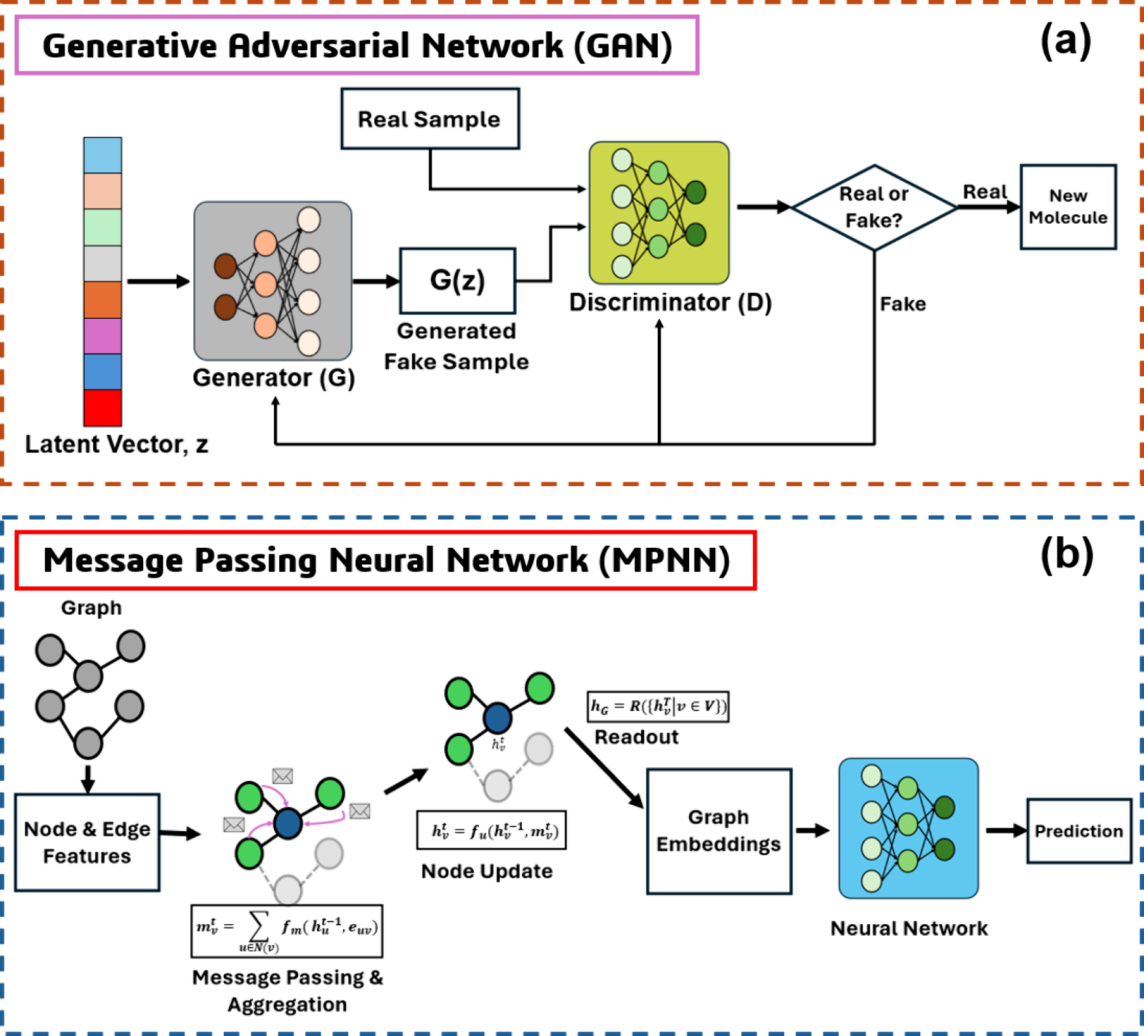
Schematic representation of the architecture of (a) GAN and (b) MPNN.

In this formulation, the generator and discriminator engage in a dynamic adversarial game, aiming to reach an equilibrium where the generated data distribution matches the real data distribution. The original GAN framework minimizes the Jensen-Shannon (JS) divergence^37^ between the real and generated data distributions. However, in practice, GAN often suffers from training instabilities such as mode collapse and vanishing gradients, especially when the real and generated data distributions have little overlap. These issues arise due to the properties of JS divergence. To address these limitations, we adopted the Wasserstein GAN (WGAN)^38^ framework, which replaces the JS divergence with the Wasserstein-1 distance, also known as the Earth Mover’s Distance. The Wasserstein distance works more efficiently even if the real and generated data distributions do not overlap. The WGAN training objective can be formulated as shown in eq 2.

2 $$\begin{array}{l} min\\ G \end{array}\begin{array}{l} max\\ D\in D \end{array}E_{x∼p_{data}\left(x\right)}\left[D\left(x\right)\right]-E_{z∼p_{z}\left(z\right)}\left[D\left(G\left(z\right)\right)\right]$$

Here, D represents the set of 1-Lipschitz functions, a constraint enforced via a gradient penalty during training. Equations 3 and 4 represent the corresponding discriminator loss and generator loss, respectively.

3 $$L_{D}=E\left[D\left(G\left(z\right)\right)\right]-E\left[D\left(x\right)\right]+\lambda E_{\mathrm{x̂}}{\left({\left|\left|\nabla_{\mathrm{x̂}}D\left(\mathrm{x̂}\right)\right|\right|}_{2}-1\right)}^{2}$$

4 $$L_{G}=\text{−}E\left[D\left(G\left(z\right)\right)\right]$$

Here, *x̂* denotes interpolated samples between real and generated graphs, and *λ* is the gradient penalty coefficient.

In this study, we implemented a customized Graph-WGAN framework for molecular graph generation. The generator maps latent vectors to continuous graph representations and produces both adjacency tensors (for bond types) and feature tensors (for atom types). The discriminator operates directly on the graph representations using graph convolution layers, which capture both molecular topology and bond-type interactions and generate node embeddings. Graph convolution layers iteratively update each node’s features by aggregating information from its neighboring nodes and bonds. With multiple iterations, each node not only gathers features from its direct neighbors but also indirectly incorporates information from more distant nodes as those neighbors propagate their own aggregated features. A global average pooling layer was used to aggregate the node embeddings into a fixed-size molecular embedding, which was further passed through multiple dense layers, leading to a single scalar output that reflects the validity score of each molecule.

We performed the GAN training using the Adam optimizer, with a learning rate set to 5 × 10^–5^ for both the generator and discriminator networks. A batch size of 32 was used, and training was conducted for 20 epochs. The generator network consisted of three fully connected layers with 128, 256, and 512 neurons, respectively, each followed by a *tanh* activation function and dropout regularization. Concurrently, along with multiple graph convolution layers, the discriminator network was structured with three fully connected layers containing 512, 256, and 128 neurons, respectively, each followed by ReLU activation functions and dropout layers to enhance generalization. A final dense layer in the discriminator produced a scalar output representing the validity score of the input molecular graph. Softmax activation was applied at the output of the generator to ensure that generated adjacency matrices and feature tensors represented valid probability distributions over bond types and atom types, respectively. Throughout the training, the gradient penalty was incorporated to maintain training stability.

### 2.3 MPNN Architecture

MPNN is a graph-based neural network designed to operate on structured data, specifically graphs, by directly utilizing atomic and bonding information to predict molecular properties. Figure 2b represents the architecture of an MPNN model. The MPNN model consisted of several sequential message passing layers, each comprising two key operations: 1) Message function: where each node aggregates information from its neighboring nodes and bonds; 2) Update function: where each node updates its feature vector based on the aggregated information. Formally, at each layer *t*, the operations can be written as eqs 5 and 6.

5 $$m_{v}^{\left(t\right)}=\sum_{u\in N\left(v\right)}f_{m}\left(h_{u}^{t-1},e_{uv}\right)$$

6 $$h_{v}^{\left(t\right)}=f_{u}\left(h_{v}^{\left(t-1\right)},m_{v}^{\left(t\right)}\right)$$

Here, *h*_*v*_^(*t*)^ denotes the feature vector of node *v* at iteration *t*, *e_uv_* denotes the bond features between nodes *u* and *v*, and *N­(v)* represents the neighboring nodes of *v*. After several rounds of message passing, a readout function was applied, which aggregated node features into a fixed-size molecular representation using a global sum pooling operation. This pooled representation was then passed through multiple fully connected layers to predict the desired molecular properties.

In this study, an MPNN framework was developed to predict key electrochemical properties of molecules, including the standard enthalpy of formation (Δ*H*_*f*_^0^), GW HOMO energy (*ε*_*HOMO*_^*GW*^), and GW LUMO energy (*ε*_*LUMO*_^*GW*^). The standard enthalpy of formation data used for MPNN training was obtained from Narayanan et al.^34^ for the QM9 dataset that used the G4MP2 method. The frontier orbital energies (HOMO/LUMO) data were taken from Fediai et al.^35^, calculated using the GW methodology. The MPNN was trained using the stochastic gradient descent (SGD) optimizer with a learning rate of 0.05, batch size of 32, and mean squared error (MSE) as the loss function. Training was conducted for 100 epochs. The molecular embedding, obtained after message passing and pooling operation, was further passed through a sequence of dense layers with hidden units [2048, 1024, 256, 128, 64], each with ReLU activations. For final property prediction, an additional branch was created using dense layers [64, 32, 16] ending in a single output neuron for the property prediction. Two different MPNN models were trained: 1) single target regression model for Δ*H*_*f*_^0^ prediction and 2) multi-target regression model for *ε*_*HOMO*_^*GW*^ and *ε*_*LUMO*_^*GW*^ predictions.

### 2.4 DFT Calculations

DFT simulations were employed to compute accurate electrochemical and thermodynamic properties for molecules that were pre-screened based on MPNN-predicted values. These properties included frontier orbital energies (HOMO and LUMO), total electronic energy, Gibbs free energy, standard enthalpy of formation, and redox potential. The goal of the DFT calculations was to provide high-fidelity reference data for evaluating and validating the performance of the deep learning models, as well as to characterize promising candidate molecules for electrolyte applications further.

All density functional theory (DFT) calculations were performed using Gaussian 16.^39^ For each molecule, geometry optimizations were carried out, followed by vibrational frequency analyses to confirm that the optimized structures correspond to true minima on the potential energy surface, as indicated by the absence of imaginary frequencies. Calculations were performed using the B3LYP exchange–correlation functional in combination with both the 6-31+G* and aug-cc-pVTZ basis sets, with all reported energetic properties evaluated consistently at each level of theory.

The use of two basis sets enabled a systematic assessment of basis-set dependence in the computed properties. The 6-31+G* basis set provides an adequate description of polarization and diffuse effects at moderate computational cost, while the larger aug-cc-pVTZ basis set offers a more complete representation of the electronic wavefunction, particularly for systems involving delocalized charge, radicals, or redox processes. Solvent effects were included using the SMD implicit solvation model, with acetone as the solvent (dielectric constant ε = 20.7), to approximate the experimental environment.

We used a thermodynamic cycle approach to calculate the oxidation and reduction potentials, considering the solvent effect and geometric relaxation. Figure 3 represents the thermodynamic cycle for the redox reaction of solvent molecules. Based on the cycle, the oxidation and reduction potentials are calculated using eqs 7 and 8.

7 $$E_{ox}\left(vs\text{.}\;Li/Li^{+}\right)=\frac{\left(\Delta G_{e}+\Delta G_{sol}^{0}\left(M^{+}\right)-\Delta G_{sol}^{0}\left(M\right)\right)}{F}-1.40$$

8 $$E_{red}\left(vs\text{.}\;Li/Li^{+}\right)=\frac{\left(\Delta G_{ea}+\Delta G_{sol}^{0}\left(M^{-}\right)-\Delta G_{sol}^{0}\left(M\right)\right)}{F}-1.40$$

Here, Δ*G_e_* and Δ*G_ea_* are the free energies of ionization in the gas phase, Δ*G*_*sol*_^0^(*M*) is the solvation energy of the species M, Δ*G*_*sol*_^0^(*M* ^+^) is the solvation free energy of the oxidized M, and Δ*G*_*sol*_^0^(*M* ^–^) is the solvation free energy of the reduced M. To obtain potentials relative to the Li reference electrode, the electrode potential of Li/Li^+^ (1.4 V) was subtracted from the computed oxidation and reduction potentials.

**Figure 3:**
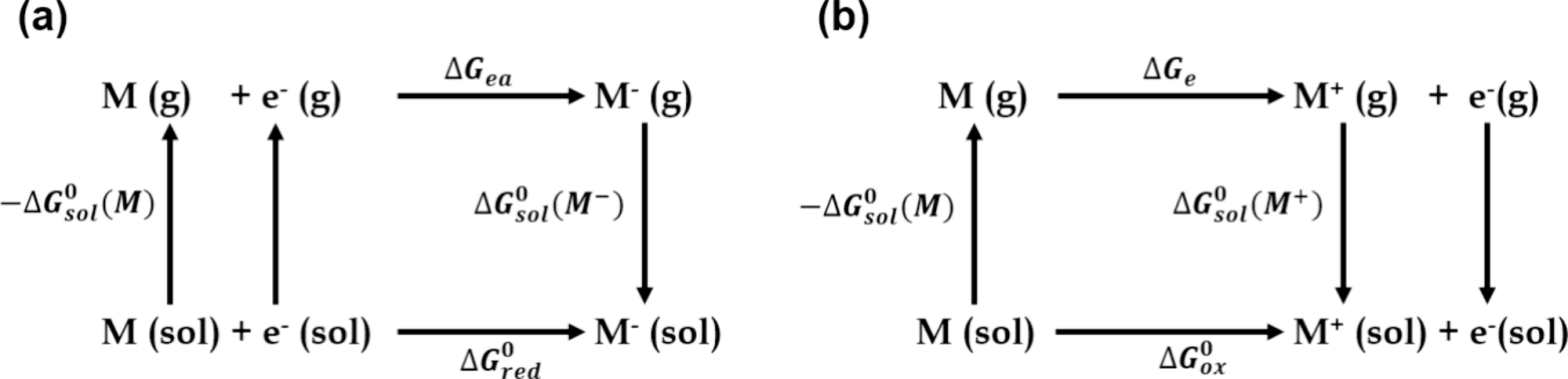
Thermodynamic cycle for the (a) reduction and (b) oxidation potential calculations.

## 3 Results and Discussions

### 3.1 Generation of New Electrolyte Molecules

We employed our developed GAN model to generate novel molecules for potential electrolyte solvent applications. The molecular generation process incorporated several critical evaluation criteria, including validity, structural uniqueness, novelty, and diversity across chemical space. Following model training, we conducted iterative molecular generation to maximize novel candidate discovery. In each generation cycle, the model produced 50,000 molecular structures, which were subsequently encoded as SMILES strings. We used RDKit to sanitize and validate these molecular structures. This involved verifying correct valence states, eliminating any disconnected molecular structures, and ensuring aromatic bonds were properly identified. The generation process was continued until reaching saturation, defined as fewer than 10 novel, unique molecules being produced across 10 consecutive iterations. At this point, it was determined that the most accessible chemical space had been explored. The validity of the generated molecules was critical for this work. Molecules that failed the sanitization step were removed from further analysis. On average, about 85% of the molecules generated in each iteration passed all chemical validity checks. This high percentage indicates that our GAN model learned the chemical rules successfully and was able to generate structures that are correct in terms of chemical perspective. Uniqueness was another important aspect of the evaluation. For each set of valid molecules, we calculated the number of distinct structures. Approximately 98% of the valid molecules were unique. The high uniqueness rate confirms that the model consistently produced novel chemical structures across iterations, rather than replicating existing outputs. Such diversity is important for covering broad areas of the chemical space. Additionally, we examined the novelty of the generated molecules. Novelty was measured by comparing the valid generated molecules to those in the training dataset. Molecules that were not present in the original training data were considered novel. About 20% of the valid molecules fell into this category. This suggests that the GAN was not just memorizing known structures but was capable of generating new and unexplored molecular candidates, which is essential for the discovery of new electrolyte materials. During the generation process, molecules were iteratively selected based on validity, uniqueness, and novelty. At each iteration, the newly generated molecules were compared with those from all previous iterations, and any duplicates were removed. Consequently, only molecules that were unique to the current iteration were retained. As the number of iterations increased, the likelihood of obtaining novel molecules progressively decreased, since many possible candidates had already appeared in earlier iterations. Finally, a set of approximately 30,000 novel molecules was obtained that contained a wide variety of molecular fragments. Examples include >C=O groups, −C≡N groups, ether linkages like −C–O–C–, and so on.

To further investigate the diversity and distribution of the generated molecules, we performed a t-SNE (t-Distributed Stochastic Neighbor Embedding) analysis.^40^ Molecular graphs from both the GAN-generated molecules and the molecules from GDB-11 were encoded and projected into a two-dimensional space. This visualization enabled a direct comparison of how the generated molecules were positioned relative to the known chemical space. Figure 4 presents the t-SNE visualization of the generated molecules relative to the molecules from GDB-11, where each point corresponds to a single molecule. The plot indicates that the majority of generated molecules occupy the core chemical space of the training dataset molecules. The generated molecules are also observed to be widely distributed across the chemical space, without forming a tight cluster. Furthermore, the dispersion of generated molecules across the entire feature space highlights the model’s ability to produce novel yet chemically valid structures, which confirms the effectiveness of the generative framework for expanding the molecular design space. Overall, the t-SNE analysis highlights that our GAN achieved a balanced generation strategy by capturing the general structural patterns present in the data. This reflects the ability of the generator to propose molecules that go beyond familiar patterns and venture into less explored areas of chemical space, and demonstrates the diversity of the generated samples.

**Figure 4:**
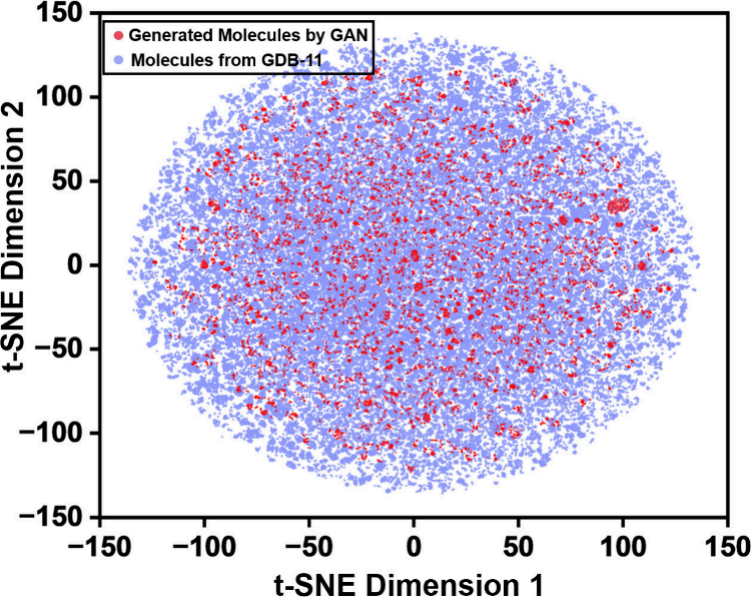
t-SNE projection of molecular graphs comparing the distribution of the training dataset and GAN-generated molecules in chemical space. The cyan dots represent molecules sampled from the training dataset, while the red dots indicate molecules generated by the GAN model. The overlapping and dispersed patterns of generated molecules suggest that the GAN successfully captured the underlying distribution of chemical space while also exploring novel regions beyond the molecules it was trained on.

To evaluate the synthetic feasibility of the molecules generated by the generative model, we assessed their Synthetic Accessibility (SA) scores using the RDKit implementation. The SA score provides an empirical estimate of how readily a molecule can be synthesized, based on structural complexity, fragment contributions, and the presence of challenging functional motifs. Our analysis shows that a substantial fraction of the generated molecules falls within an SA score range of approximately 2–5 (Figure S1), which is generally considered indicative of good synthetic accessibility.^41^ This suggests that, despite being novel, many of the generated structures are not overly complex or synthetically unrealistic. Furthermore, the overall SA score distribution of the generated molecules is comparable to that observed for known, synthesizable compounds in widely used chemical databases. This finding indicates that our generative model is capable of proposing chemically reasonable and practically feasible candidates.

### 3.2 Prediction of Properties of Generated Molecules with MPNN

Following the successful generation of novel molecules through the GAN model, the next phase of this study focused on evaluating their fundamental electrochemical properties to assess their suitability as potential battery electrolyte candidates. For this purpose, an MPNN model was implemented and trained on the well-established QM9 dataset.^34,35^ The QM9 dataset is a widely used benchmark in molecular ML studies and consists of over 133,000 organic molecules with up to nine heavy atoms. It includes a diverse range of chemical structures, along with computed properties from quantum chemistry calculations. From this dataset generated using, we selected three important properties related to the stability and electrochemical behavior of molecules—the standard enthalpy of formation, HOMO energy, and LUMO energy.

The dataset was divided into 80% for training, 10% for validation, and 10% for testing. Two different regression models were used in this study. The first was a single-target MPNN model, which was trained to predict the standard enthalpy of formation, Δ*H*_*f*_^0^. The second was a multi-target regression model, designed to predict both HOMO, *ε*_*HOMO*_^*GW*^ and LUMO energy, *ε*_*LUMO*_^*GW*^ simultaneously. The performance of the trained MPNN model was evaluated quantitatively using standard regression metrics such as mean absolute error (MAE) and coefficient of determination (R^2^ score) on both validation and test sets derived from QM9.

Figure 5 shows the parity plots for the prediction values. For Δ*H*_*f*_^0^, the MPNN model performed exceptionally well. It achieved R^2^ scores of 96.78%, 96.77% and 96.82% for the training, validation and testing sets, respectively. The corresponding MAE values were 7.66, 7.63, and 7.68 kcal/mol, respectively. For *ε*_*HOMO*_^*GW*^, the model also showed strong performance. The R^2^ scores reached 89.69% for the training set, 88.57% for the validation set, and 88.33% for the testing set. The corresponding MAE values were 0.121, 0.127, and 0.128 eV, respectively. Similarly, for *ε*_*LUMO*_^*GW*^, the model achieved R^2^ scores of 94.41%, 93.87%, and 93.81% for training, validation, and testing sets, respectively. The associated MAE values were 0.166, 0.172, and 0.170 eV. Following this validation, the trained MPNN was used to predict these properties of the 30,000 novel molecules generated by the GAN model. The distribution of the predictions for generated molecules was illustrated using violin plots in Figure 6. It was observed that the Δ*H*_*f*_^0^ of the generated molecules ranges from approximately −220 to 130 kcal/mol. The range of HOMO and LUMO energies was found to be approximately −7.2 to −4.1 eV and −4.2 to −1 eV, respectively. These predictions enabled an initial screening step to identify candidates meeting the desired electrochemical criteria.

**Figure 5:**
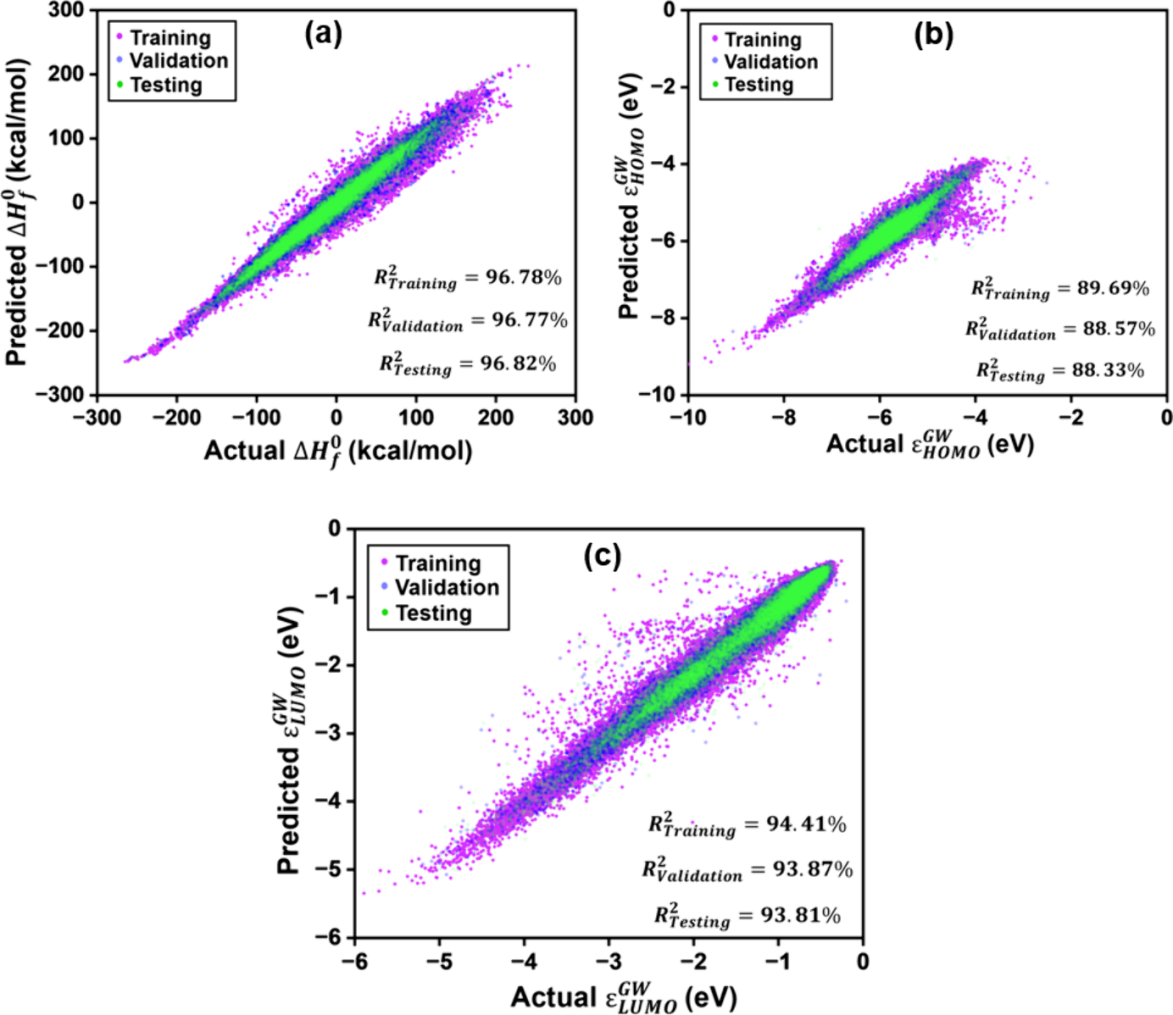
Parity plots showing the comparison of actual vs MPNN predicted values for (a) Δ*H*_*f*_^0^, (b) *ε*_*HOMO*_^*GW*^, and (c) *ε*_*LUMO*_^*GW*^.

**Figure 6:**
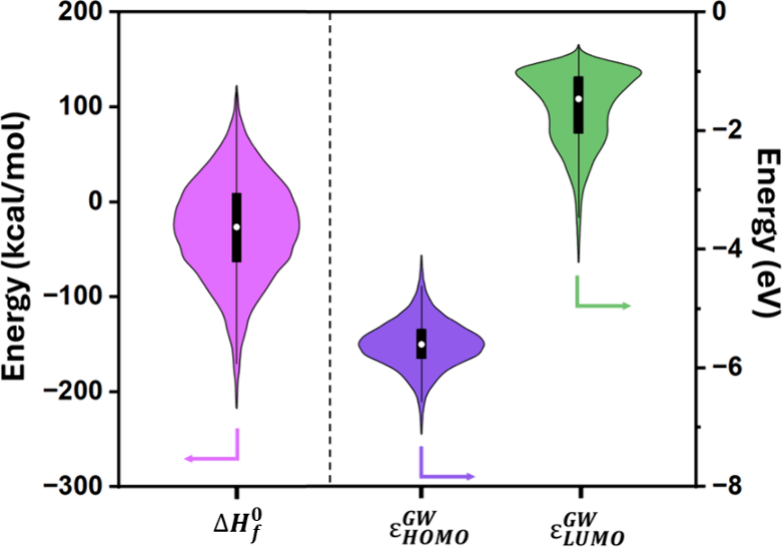
The violin plot, representing the distribution of the prediction values of Δ*H*_*f*_^0^, *ε*_*HOMO*_^*GW*^, and *ε*_*LUMO*_^*GW*^ for the generated molecules.

A set of threshold values was applied to filter the generated molecules. Initially, all the 30000 generated molecules were screened based on the standard enthalpy of formation values. Only molecules with negative formation enthalpies were considered for further evaluation. This filtering step excluded approximately 9300 generated structures. Next, another screening was conducted for the LUMO energy values, where the threshold was set to be greater than −2 eV. This step eliminated 7625 molecules that did not satisfy the criteria. Finally, we filtered the remaining molecules to include only those with HOMO energies below −6.65 eV and obtained a set of 50 candidates (Figure S2). We proceeded with further analysis of these molecules, as reported in the next section.

### 3.3 DFT Calculations

After generating and screening molecules using GAN and MPNN models, a set of 50 electrolyte candidates was selected for further detailed analysis using DFT calculations. The primary objective of this step was to obtain accurate values for standard enthalpy of formation, frontier orbital energies (HOMO and LUMO), and oxidation and reduction potentials to assess their suitability as electrolyte candidates.

#### 3.3.1 Standard Enthalpy of Formation

To evaluate the stability and feasibility of the synthesis of the new molecules, it is crucial to calculate the standard enthalpy of formation of the molecules in reference to their elementary phases. In this scenario, a negative standard enthalpy of formation indicates a thermodynamic stability of the molecule. As previously mentioned, the large set of new electrolyte candidates was downsized with their MPNN-predicted standard enthalpy of formation and frontier energies. A single target MPNN regression model was trained to predict the standard enthalpies of formation for the generated molecules by GAN. The MPNN predicted standard enthalpy of formation of the downsized molecules was further validated using G4MP2 calculations. As the energies of elemental reference states (C, H, O, N, F), the G4MP2 energies of isolated atoms in their ground states were utilized. The final 50 molecules were first subjected to geometric optimization. Following the optimization, vibrational frequency calculations were carried out to confirm the structural stability. Molecules with no imaginary frequencies were identified as being in the true minima on the potential energy surface. After confirming the stability of the geometries, we calculated the Gibbs free energies for all molecules. The detailed procedure for calculating the enthalpy of formation is provided in ref 34.

We found that among 50 candidates, 24 molecules exhibit a positive standard enthalpy of formation. These molecules were excluded from subsequent calculations to ensure the selection of thermodynamically stable candidates. Figure 7 depicts the comparative evaluation of our DFT calculated standard enthalpy of formation for the remaining 26 molecules with MPNN prediction. Despite some noticeable spread between the predicted and calculated quantities, the overall finding indicates that the MPNN model captures the general trend of DFT calculations reasonably well. This clearly illustrates the effectiveness of our MPNN model. While there is room for improvement, the predictions are sufficiently accurate to demonstrate the model’s predictive capability and to provide reasonable estimates of the standard enthalpy of formation. In Figure S3, we represented all the final 26 candidates and assigned individual indices M01-M26 for each molecule for the convenience of the representation. One interesting fact was noticed that among them, the majority of all the molecules contain a nitrile group in their structure. Among the generated molecules, only about 5% contains the nitrile functional group, as shown in the t-SNE plot provided in Figure S4. However, after predicting their HOMO, LUMO, and formation enthalpy using the trained MPNN model and applying our screening criteria, we observed that most of the top-ranked candidates fall into the nitrile class. This motivated us to further analyze other key electrochemical properties that highly influence the performance of the battery system and whether the nitrile group has any exceptional characteristics that affect them.

**Figure 7:**
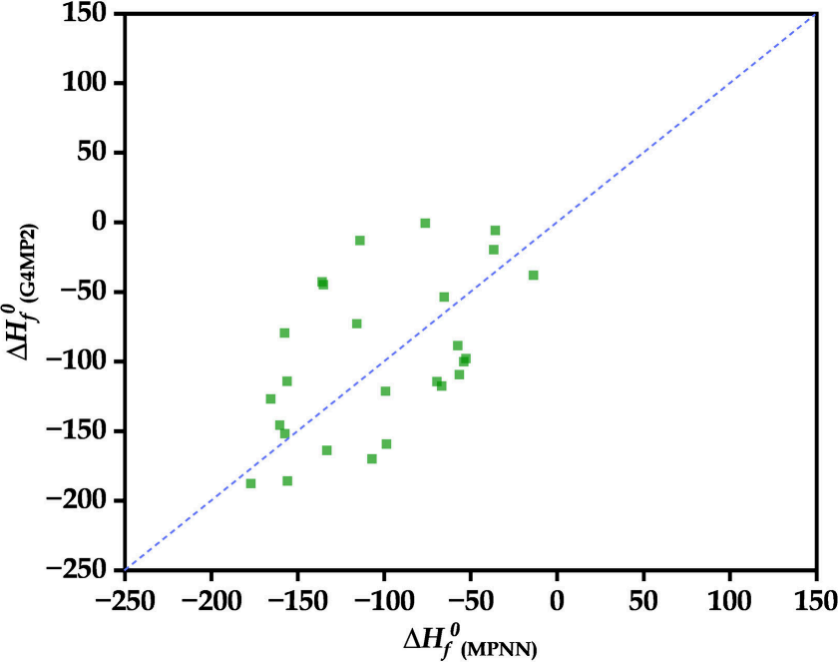
MPNN-predicted property standard enthalpy of formation plotted against their G4MP2-calculated counterparts, with the diagonal indicating perfect agreement.

#### 3.3.2 HOMO-LUMO Energies

Along with the molecular structures, the calculated frontier orbital energies are shown in Figure S3. A comparison between the calculated HOMO and LUMO energies from DFT and those predicted by the MPNN model is presented in Figure 8. The selection of candidate molecules from MPNN predictions was based on specific screening criteria applied to the MPNN predictions. These criteria set the HOMO energies to be lower than −6.65 eV and the LUMO energies to be higher than −2.0 eV. This window was chosen to ensure both oxidative and reductive stability for potential electrolyte candidates. However, systematic deviations were observed between the MPNN predictions and our DFT results. Our DFT calculated HOMO energies were consistently lower than those predicted by the MPNN. On average, the HOMO levels showed a downward shift of approximately 1.5 eV. In contrast, the LUMO energies calculated by DFT were higher than the MPNN predictions, with an average upward deviation of about 0.90 eV. These systematic shifts are expected, given that MPNN was trained on QM9 data computed with the eigenvalue self-consistent GW method using aug-cc-DZVP and aug-cc-TZVP basis sets. In contrast, we used the B3LYP functional with the 6-31+G* and aug-cc-pVTZ basis sets for DFT calculations. Similar deviations between GW and PBE functional results have also been reported previously.^35^ Nevertheless, the observed trends remained consistent, and all molecules still satisfied the screening requirements after DFT validation. In addition, comparison of the DFT results obtained using the 6-31+G* and aug-cc-pVTZ basis sets shows that both basis sets are in close agreement for both HOMO and LUMO energies. The average differences between the two basis sets are relatively small, with values of approximately 0.09 eV for HOMO energies and 0.13 eV for LUMO energies. The relative trends across the molecular set remain consistent between the two calculations. These observations indicate that the relatively small 6-31+G* basis set is sufficient to accurately describe the electronic and electrochemical properties at a substantially lower computational cost compared to the aug-cc-pVTZ basis set.

**Figure 8:**
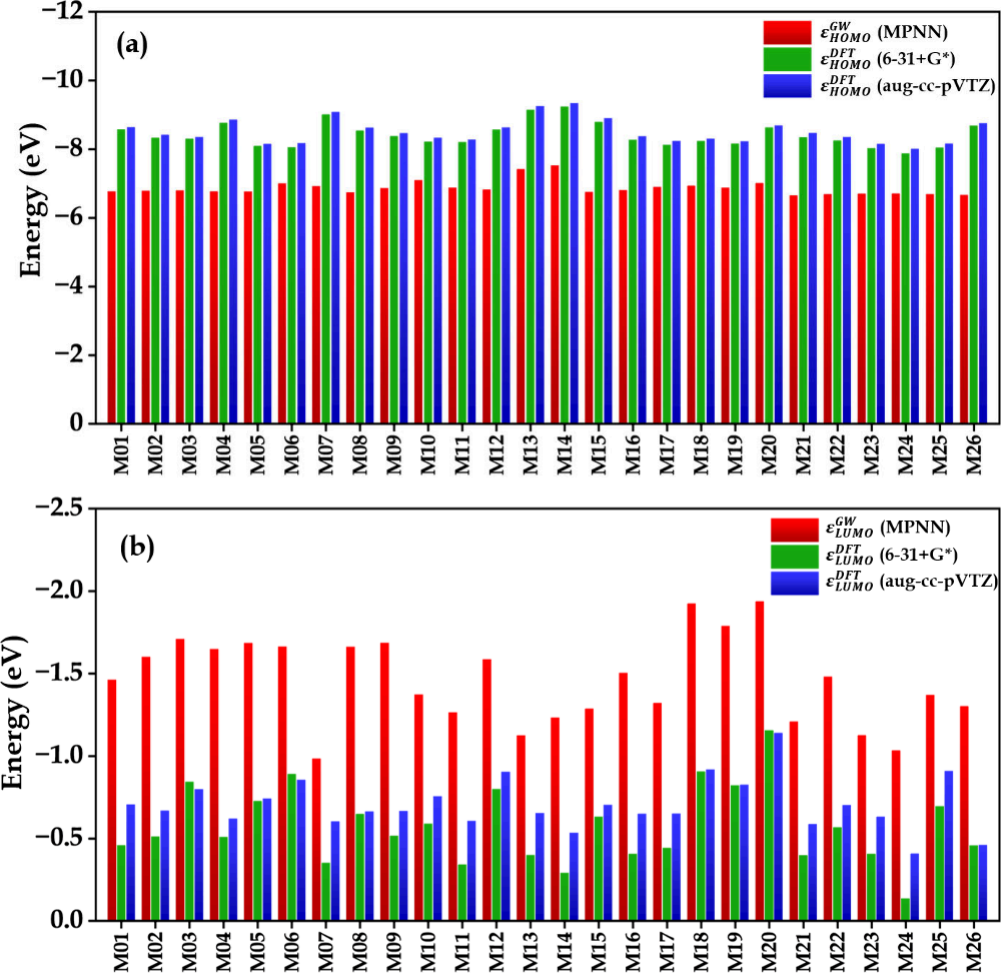
Comparison of frontier orbital energies predicted by the MPNN and calculated using DFT. (a) HOMO energies and (b) LUMO energies for molecules M01–M26. DFT values were obtained at the B3LYP level using the 6-31+G* and aug-cc-pVTZ basis sets.

#### 3.3.3 Oxidation/Reduction Potentials

The performance of an electrolyte highly depends on its oxidation and reduction potentials. Molecules with high oxidation potentials are less likely to decompose at high-voltage cathode interfaces, which ensures oxidative stability during operation. On the other hand, molecules with suitably low reduction potentials can resist breakdown at the anode interface and maintain stability against reductive decomposition. For this reason, electrolytes should be carefully selected based on these properties to ensure stable performance within the desired voltage range. With this in mind, we further continued our study by calculating the oxidation and reduction potentials of the final set of 26 generated molecules to investigate their suitability as electrolyte candidates. To validate our DFT calculations with the literature, initially, we calculated the oxidation potentials for some known and widely used organic electrolyte solvent molecules—1,2-dimethoxyethane (DME), 1,3-dioxolane (DOL), and ethylene carbonate (EC). Our study determined the oxidation potentials of DME, DOL, and EC to be approximately 5.47, 5.43, and 6.67 V vs. Li/Li^+^, respectively. These findings are in good agreement with the previously reported studies, where the oxidation potentials of DME (5.66 V)^42^, DOL (5.74 V)^43^, and EC (7.01 V)^44^ closely align with our calculated values. According to previous studies, the oxidation potential of commonly used electrolyte solvents typically falls within the range of approximately 4 to 6 V vs. Li/Li^+^,^43^ which is generally accepted as a practical criterion for stable electrolyte molecules. To assess whether our generated molecules meet or exceed this benchmark, we calculated their oxidation potentials using the DFT-based thermodynamic cycle method. The results revealed that all 26 molecules exhibited oxidation potentials in the range of approximately 5.6 to 6.9 V vs. Li/Li^+^ (Figure 9). This range not only satisfies but slightly exceeds the typical values reported for commercial electrolyte solvents.

**Figure 9:**
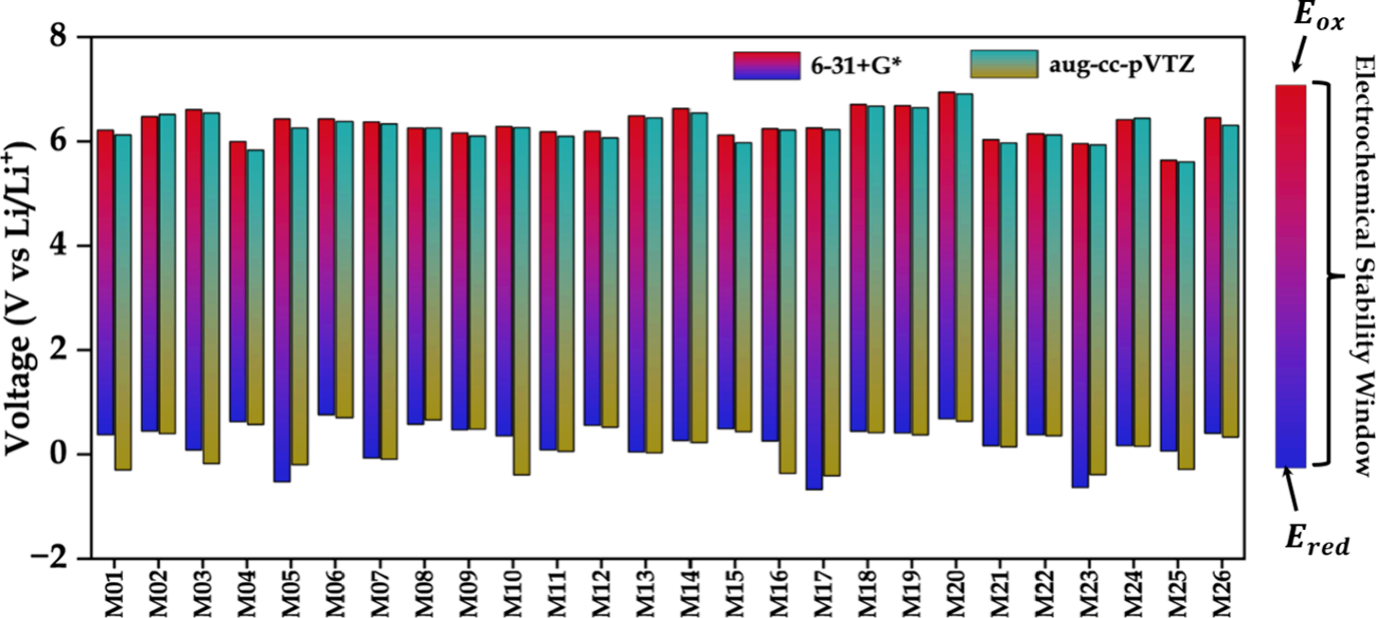
Oxidation and reduction potentials calculated for the final 26 screened molecules.

In addition to oxidation potential, the reduction potentials of all 26 molecules were calculated to assess their stability against reductive decomposition. The reduction potentials of the 26 molecules were calculated and found to range from −0.68 V to 0.75 V vs. Li/Li^+^. According to previous studies on electrolyte species, such as the work by Han et al.^45^, though common electrolyte isolated molecules possess negative reduction potential values, reduction potentials less than 1 V vs. Li/Li^+^ are considered suitable for lithium metal batteries. In this context, the majority of our molecules fall within or near the practical electrochemical window. Molecules with slightly positive reduction potentials are likely to show moderate reactivity, which can be advantageous for forming stable solid electrolyte interphases if required. On the other hand, molecules with negative reduction potential indicate inherent stability against unwanted reductive decomposition. Comparison of the oxidation and reduction potentials calculated using the 6-31+G* and aug-cc-pVTZ basis sets shows close agreement across the molecules. The electrochemical stability windows predicted by the two basis sets differ only slightly, with an average difference of approximately 0.06 V for the oxidation potentials and 0.16 V for the reduction potentials, while maintaining consistent relative trends among the candidate molecules. Consequently, the resulting electrochemical stability windows derived from the two basis sets are also very similar. Together, these results suggest that our proposed 26 final candidate molecules possess superior electrochemical stability, which makes them suitable candidates for further consideration as electrolyte components.

## 4 Conclusion

In this study, we aimed to discover novel electrolyte molecules for alkali metal batteries by leveraging Gen AI and graph-based ML algorithms. Our study began with the development of a GAN framework trained on graph representations of molecular structures obtained from a subset of the GDB-11 database. The molecular structures, represented as SMILES strings in the dataset, were processed using Python’s RDKit package to extract atomic and bond-level information. We found that our GAN was able to successfully generate approximately 30,000 novel and valid molecules that retained the diversity and general structural motifs of the training data, which was further confirmed by t-SNE analysis. Subsequently, we extended our investigation by training an MPNN model on the QM9 dataset using molecular structural information as descriptors to predict key electrochemical properties, including the standard enthalpy of formation, HOMO energy, and LUMO energy. Our developed MPNN model achieved high predictive accuracies of over 96%, 88%, and 93% for these properties, respectively. We applied the trained MPNN model to the 30,000 GAN-generated molecules to predict their electrochemical properties. A stringent screening criterion, incorporating negative standard enthalpy of formation and a wide electrochemical window, was employed to downsize the candidates based on their predicted electrochemical properties. A set of 50 electrolyte candidate molecules was obtained. Finally, DFT calculations, using 6-31+G* and aug-cc-pVTZ basis sets and G4MP2 method, were performed on these final candidates to validate the predicted properties and determine their oxidation and reduction potentials. Based on the negative standard enthalpy of formation, only 26 molecules were selected as the final electrolyte candidates. Our analysis suggests that the selected final molecules exhibited favorable electrochemical characteistics, with oxidation potentials ranging from 5.6 to 6.9 V vs. Li/Li^+^ and reduction potentials ranging from −0.68 to 0.75 V vs. Li/Li^+^. Overall, this work demonstrates that the integration of GAN and MPNN models provides an effective approach for accelerating the discovery of electrolyte molecules with desirable properties for alkali metal batteries.

## Supplementary Material



## Data Availability

All datasets used and generated in this study, along with the MPNN source code, are openly available in the Zenodo repository under the DOI 10.5281/zenodo.17419502. The DFT calculations were performed using the Gaussian 16 software package (https://gaussian.com/gaussian16/).

## Notes

Distribution of the SA score of all GAN-generated molecules, screening process of the generated molecules, downselected electrolyte candidates, and their key electrochemical properties calculated with aug-cc-pVTZ basis, and t-SNE analysis of the carbonyl group-containing generated molecules (PDF)
