# Supplementary material for: Generative AI-Driven Discovery of Next-Generation Electrolytes for Alkali Metal Batteries
Source: J Chem Inf Model. 2026 Mar 13;66(7):3747–58. doi: 10.1021/acs.jcim.6c00135 (PMC13080966; doi:10.1021/acs.jcim.6c00135)
Supplement: Supplementary file 1 [file ci6c00135_si_001.pdf]

Supporting Information

of

Generative AI-Driven Discovery of Next-Generation Electrolytes for Alkali Metal Batteries

Rafiuzzaman Pritom[a] and Md Mahbubul Islam[a,*]

[a]Department of Mechanical Engineering, Wayne State University, Detroit, Michigan 48202, USA

[*]Corresponding Author: gy5553@wayne.edu

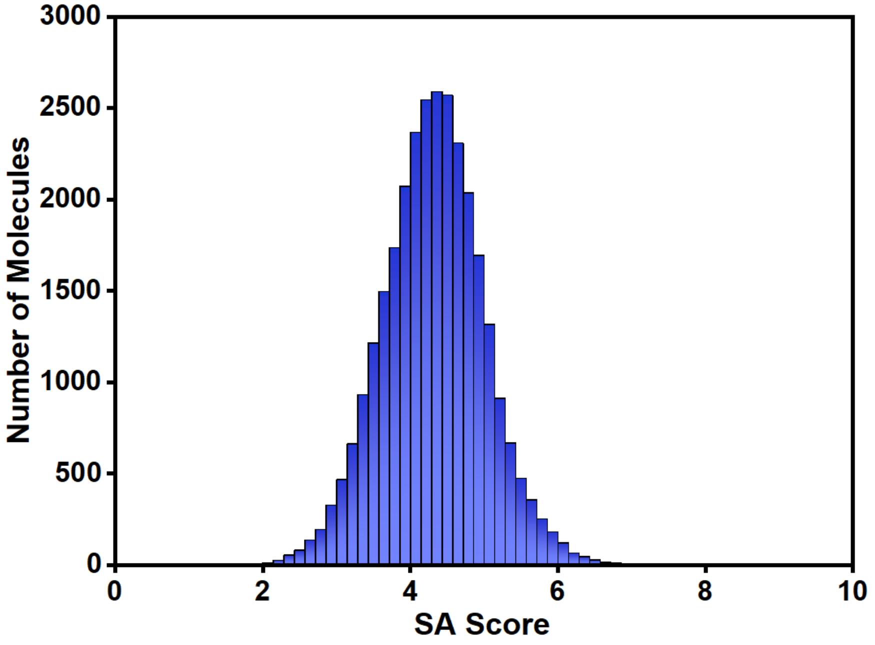

Figure S1. Distribution of the SA score of all the 30000 GAN-generated molecules

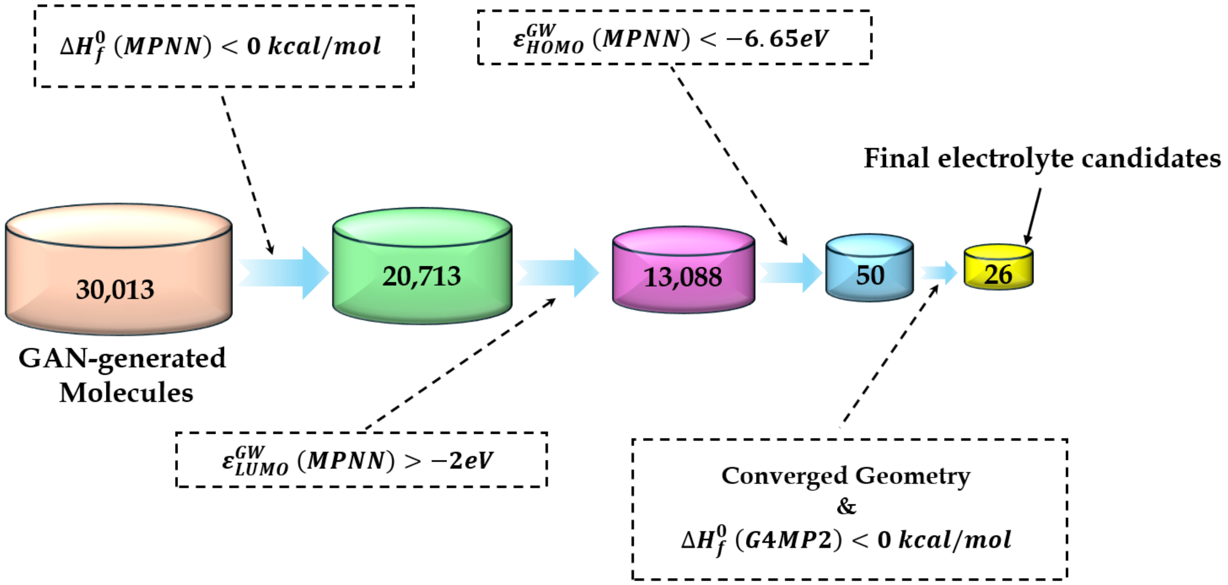

Figure S2. Screening process of the generated molecules based on MPNN prediction and DFT calculations

M01

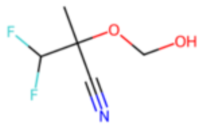

CC(C#N)(OCO)C(F)F

ΔH_f^0 : -145.98 kcal/mol

ε_{HOMO}^{DFT} : -8.64 eV

ε_{LUMO}^{DFT} : -0.70 eV

E_{ox} : 6.12 V

E_{red} : -0.30 V

M02

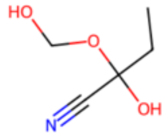

CCC(O)(C#N)OCO

ΔH_f^0 : -159.66 kcal/mol

ε_{HOMO}^{DFT} : -8.42 eV

ε_{LUMO}^{DFT} : -0.67 eV

E_{ox} : 6.52 V

E_{red} : 0.39 V

M03

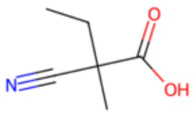

CCC(C)(C#N)C(=O)O

ΔH_f^0 : -114.55 kcal/mol

ε_{HOMO}^{DFT} : -8.36 eV

ε_{LUMO}^{DFT} : -0.80 eV

E_{ox} : 6.54 V

E_{red} : -0.18 V

M04

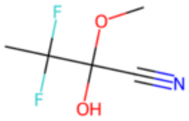

COC(O)(C#N)C(C)(F)F

ΔH_f^0 : -152.08 kcal/mol

ε_{HOMO}^{DFT} : -8.86 eV

ε_{LUMO}^{DFT} : -0.62 eV

E_{ox} : 5.83 V

E_{red} : 0.57 V

M05

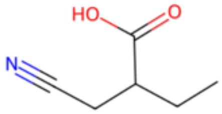

CCC(CC#N)C(=O)O

ΔH_f^0 : -117.71 kcal/mol

ε_{HOMO}^{DFT} : -8.15 eV

ε_{LUMO}^{DFT} : -0.74 eV

E_{ox} : 6.25 V

E_{red} : -0.20V

M06

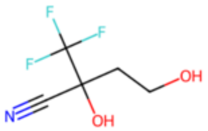

N#CC(O)(CCO)C(F)(F)F

ΔH_f^0 : -188.04 kcal/mol

ε_{HOMO}^{DFT} : -8.18 eV

ε_{LUMO}^{DFT} : -0.86 eV

E_{ox} : 6.38 V

E_{red} : 0.70 V

M07

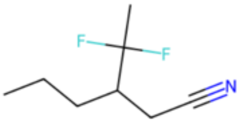

CCCC(CC#N)C(C)(F)F

ΔH_f^0 : -0.76 kcal/mol

ε_{HOMO}^{DFT} : -9.08 eV

ε_{LUMO}^{DFT} : -0.60 eV

E_{ox} : 6.33 V

E_{red} : -0.10 V

M08

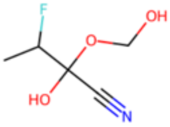

CC(F)C(O)(C#N)OCO

ΔH_f^0 : -186.22 kcal/mol

ε_{HOMO}^{DFT} : -8.63 eV

ε_{LUMO}^{DFT} : -0.66 eV

E_{ox} : 6.25 V

E_{red} : 0.66 V

M09

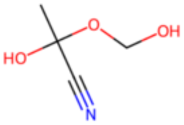

CC(O)(C#N)OCO

ΔH_f^0 : -170.29 kcal/mol

ε_{HOMO}^{DFT} : -8.46 eV

ε_{LUMO}^{DFT} : -0.67 eV

E_{ox} : 6.10 V

E_{red} : 0.48 V

M10

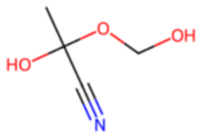

N#CC(CCO)C(F)(F)F

ΔH_f^0 : -127.18 kcal/mol

ε_{HOMO}^{DFT} : -8.33 eV

ε_{LUMO}^{DFT} : -0.75 eV

E_{ox} : 6.26 V

E_{red} : -0.40 V

M11

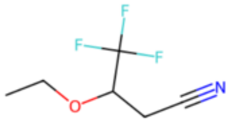

CCOC(CC#N)C(F)(F)F

ΔH_f^0 : -114.45 kcal/mol

ε_{HOMO}^{DFT} : -8.28 eV

ε_{LUMO}^{DFT} : -0.61 eV

E_{ox} : 6.09 V

E_{red} : 0.05 V

M12

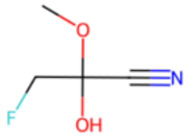

COC(O)(C#N)CF

ΔH_f^0 : -121.66 kcal/mol

ε_{HOMO}^{DFT} : -8.63 eV

ε_{LUMO}^{DFT} : -0.90 eV

E_{ox} : 6.06 V

E_{red} : 0.52 V

M13

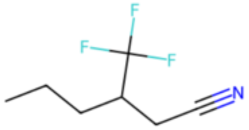

CCCC(CC#N)C(F)(F)F

ΔH_f^0 : -45.05 kcal/mol

ε_{HOMO}^{DFT} : -9.25 eV

ε_{LUMO}^{DFT} : -0.65 eV

E_{ox} : 6.45 V

E_{red} : 0.03 V

M14

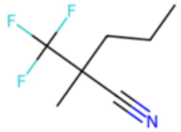

CCCC(C)(C#N)C(F)(F)F

ΔH_f^0 : -42.92 kcal/mol

ε_{HOMO}^{DFT} : -9.34 eV

ε_{LUMO}^{DFT} : -0.53 eV

E_{ox} : 6.54 V

E_{red} : 0.22 V

M15

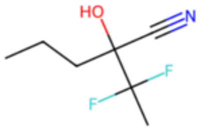

CCCC(O)(C#N)C(C)(F)F

ΔH_f^0 : -73.07 kcal/mol

ε_{HOMO}^{DFT} : -8.90 eV

ε_{LUMO}^{DFT} : -0.70 eV

E_{ox} : 5.97 V

E_{red} : 0.43 V

M16

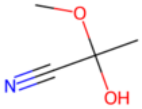

COC(C)(O)C#N

ΔH_f^0 : -100.27 kcal/mol

ε_{HOMO}^{DFT} : -8.37 eV

ε_{LUMO}^{DFT} : -0.65 eV

E_{ox} : 6.22 V

E_{red} : -0.37 V

M17

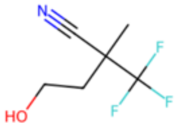

CC(C#N)(CCO)C(F)(F)F

ΔH_f^0 : -79.76 kcal/mol

ε_{HOMO}^{DFT} : -8.24 eV

ε_{LUMO}^{DFT} : -0.65 eV

E_{ox} : 6.23 V

E_{red} : -0.42 V

M18

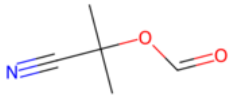

CC(C)(C#N)OC=O

ΔH_f^0 : -109.61 kcal/mol

ε_{HOMO}^{DFT} : -8.30 eV

ε_{LUMO}^{DFT} : -0.92 eV

E_{ox} : 6.67 V

E_{red} : 0.41 V

M19

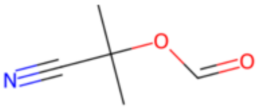

CCC(C)(C#N)OC=O

ΔH_f^0 : -98.21 kcal/mol

ε_{HOMO}^{DFT} : -8.23 eV

ε_{LUMO}^{DFT} : -0.83 eV

E_{ox} : 6.64 V

E_{red} : 0.37 V

M20

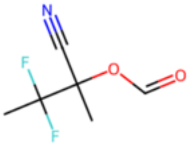

CC(F)(F)C(C)(C#N)OC=O

ΔH_f^0 : -164.14 kcal/mol

ε_{HOMO}^{DFT} : -8.69 eV

ε_{LUMO}^{DFT} : -1.14 eV

E_{ox} : 6.91 V

E_{red} : 0.63 V

M21

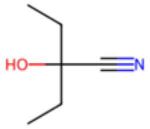

CCC(O)(C#N)CC

ΔH_f^0 : -19.82 kcal/mol

ε_{HOMO}^{DFT} : -8.47 eV

ε_{LUMO}^{DFT} : -0.59 eV

E_{ox} : 5.97 V

E_{red} : 0.14 V

M22

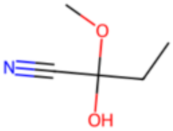

CCC(O)(C#N)OC

ΔH_f^0 : -88.90 kcal/mol

ε_{HOMO}^{DFT} : -8.36 eV

ε_{LUMO}^{DFT} : -0.70 eV

E_{ox} : 6.12 V

E_{red} : 0.35 V

M23

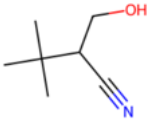

CC(C)(C)C(C#N)CO

ΔH_f^0 : -5.90 kcal/mol

ε_{HOMO}^{DFT} : -8.15 eV

ε_{LUMO}^{DFT} : -0.63 eV

E_{ox} : 5.93 V

E_{red} : -0.39 V

M24

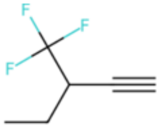

C#CC(CC)C(F)(F)F

ΔH_f^0 : -13.11 kcal/mol

ε_{HOMO}^{DFT} : -8.01 eV

ε_{LUMO}^{DFT} : -0.41 eV

E_{ox} : 6.44 V

E_{red} : 0.15 V

M25

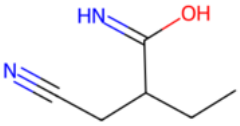

CCC(CC#N)C(=N)O

ΔH_f^0 : -38.26 kcal/mol

ε_{HOMO}^{DFT} : -8.16 eV

ε_{LUMO}^{DFT} : -0.91 eV

E_{ox} : 5.60 V

E_{red} : -0.29 V

M26

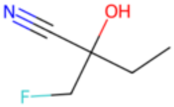

CCC(O)(C#N)CF

ΔH_f^0 : -53.95 kcal/mol

ε_{HOMO}^{DFT} : -8.75 eV

ε_{LUMO}^{DFT} : -0.46 eV

E_{ox} : 6.31 V

E_{red} : 0.33 V

Figure S3. The final 26 electrolyte candidate molecules with their DFT (aug-cc-pVTZ basis) calculated key electrochemical properties including – standard enthalpy of formation, HOMO energy, LUMO energy, oxidation potential and reduction potential

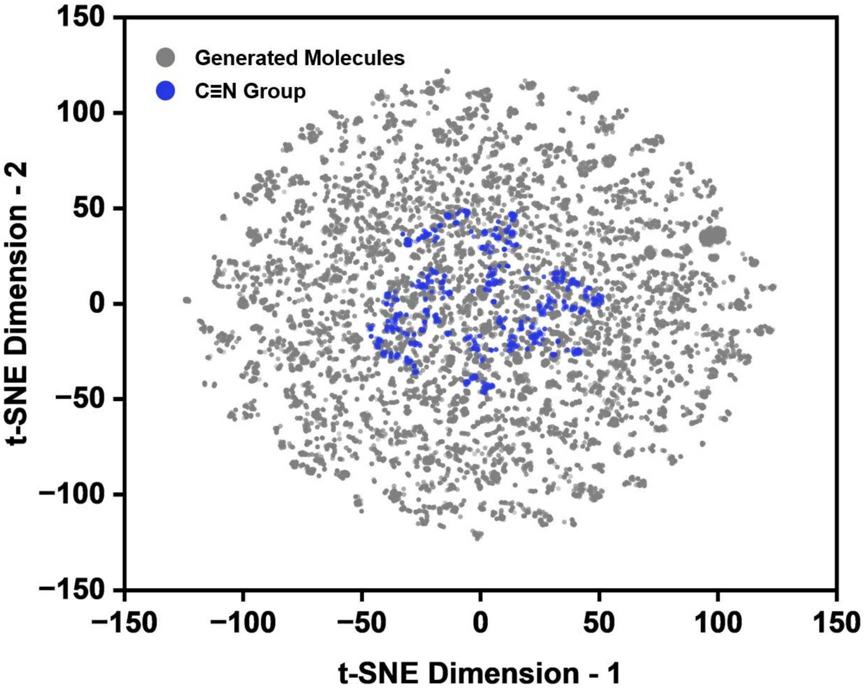

Figure S4. t-SNE analysis of the GAN-generated molecules showing the distribution of all the generated molecules and carbonyl group containing molecules
